# Family Matters: How Family Member Incarceration, and Who Was Incarcerated, is Associated with Older Adults’ Health

**DOI:** 10.1093/geroni/igaf122.3590

**Published:** 2025-12-31

**Authors:** Louisa Holaday, Albert Siu, Brie Williams, Brita Roy, Julia Wright, Pranav Gwalani, Emily Wang

**Affiliations:** Icahn School of Medicine at Mount Sinai, New York, New York, United States; Icahn School of Medicine at Mount Sinai, new york, New York, United States; University of California, San Francisco, san francisco, California, United States; NYU Langone Health, brooklyn, New York, United States; Icahn School of Medicine at Mount Sinai, new york, New York, United States; Icahn School of Medicine at Mount Sinai, New York, New York, United States; Yale University, New Haven, Connecticut, United States

## Abstract

**Intro:**

Having ever had an incarcerated family member is associated with worse health, but little is known about specifics among older adults, who have lived their entire adult lives in the era of mass incarceration.

**Methods:**

In a cross-sectional analysis of FamHIS data (2018), a nationally representative survey designed to understand family member incarceration in the United States, we used logistic regression models to examine associations with self-reported physical and mental health. Our primary exposure was any immediate family member incarceration. Secondary analyses examined relationship of incarcerated family member. Covariates were age, gender, race/ethnicity, education, income, employment, marital status, and personal incarceration history.

**Results:**

Among 1,319 subjects, of whom 63% experienced incarceration of an immediate family member, family member incarceration was independently associated with increased odds of reporting “fair” or “poor” physical and mental health (aOR 1.46; 95% Confidence interval: 1.00-2.14); P = 0.05 and aOR 2.13; 95% CI: 1.27-3.58 ; P = 0.004, respectively). This differed by relationship, with ever parental incarceration independently associated with the highest odds of “fair” or “poor” physical and mental health (aOR 1.99, 95% CI: 1.07-3.68, P = 0.03; aOR 2.95, 95% CI: 1.48-5.90 respectively, P = 0.002), followed by children and siblings, regardless of when that incarceration occurred.

**Conclusion:**

Ever incarceration of a family member was independently associated with poor health among older adults, even for those whose parent was incarcerated in the distant past. Researchers should work to understand the mechanisms behind this, and clinicians and policy makers should intervene at earlier ages to ensure all Americans can age healthfully.

